# Increased mortality risk in multiple-myeloma patients with subsequent malignancies: a population-based study in the Netherlands

**DOI:** 10.1038/s41408-022-00640-6

**Published:** 2022-03-15

**Authors:** Mirian Brink, Monique C. Minnema, Otto Visser, Mark-David Levin, Eduardus F. M. Ward Posthuma, Annemiek Broijl, Pieter Sonneveld, Marjolein van der Klift, Wilfried W. H. Roeloffzen, Matthijs Westerman, Cleo R. van Rooijen, Paul A. F. Geerts, Sonja Zweegman, Niels W. C. J. van de Donk, Avinash G. Dinmohamed

**Affiliations:** 1 Department of Research and Development, Netherlands Comprehensive Cancer Organisation (IKNL), Utrecht, The Netherlands; 2 Department of Hematology, Cancer Center, UMC Utrecht, Utrecht, The Netherlands; 3 Department of Registration, Netherlands Comprehensive Cancer Organisation (IKNL), Utrecht, The Netherlands; 4 Department of Internal Medicine, Albert Schweitzer Hospital, Dordrecht, The Netherlands; 5 Department of Internal Medicine, Reinier de Graaf Group, Delft, The Netherlands; 6 Department of Hematology, Erasmus MC Cancer Institute, Rotterdam, The Netherlands; 7 Department of Internal Medicine, Amphia Hospital, Breda, The Netherlands; 8 Department of Hematology, University Medical Center Groningen, Groningen, The Netherlands; 9 Department of Internal Medicine, Northwest Clinics, Alkmaar, the Netherlands; 10 Department of Hematology, Medical Center Twente, Enschede, The Netherlands; 11 Department of Hematology, Isala Hospital, Zwolle, The Netherlands; 12 Amsterdam UMC, Vrije Universiteit Amsterdam, Department of Hematology, Cancer Center Amsterdam, Amsterdam, The Netherlands; 13 Department of Public Health, Erasmus University Medical Center, Rotterdam, The Netherlands

**Keywords:** Cancer epidemiology, Epidemiology

The rapidly evolving treatment landscape of multiple myeloma (MM) has dramatically improved the survival of MM patients over time. However, there is also a concurrrent increase in the incidence of subsequent primary malignancies (SPMs) [1–3]. Risk factors associated with SPMs in MM encompass treatment with alkylating agents or immunomodulatory drugs (IMiDs) [1, 4–6], environmental exposures, genetic susceptibility to cancer, or combinations of these risk factors [7, 8]. As MM survivorship is expected to increase, it is vital for clinicians to know how these risk factors influence SPM development in MM patients. Therefore, this nationwide, population-based study aimed to complement and extend the data on SPMs among MM patients in the Netherlands, assessing prior malignancy diagnoses (PMDs) as a potential proxy for genetic susceptibility to cancer, as well as treatment-related factors. Besides, we assessed whether SPM development was associated with higher mortality risk.

From the nationwide Netherlands Cancer Registry (NCR), we identified all adult (≥18 years) patients with MM diagnosed between January 1, 1994 and December 31, 2013. PMDs (diagnosed between January 1, 1989 and December 31, 2013) and SPMs (diagnosed between January 1, 1994 and December 31, 2018) were identified by cross-linkage within the NCR. This period selection allowed at least five years of follow-up to capture PMDs and SPMs before and after MM diagnosis, respectively. Benign, borderline, in situ tumors, and basal-cell carcinomas (BCCs) were excluded. Furthermore, patients diagnosed with MM at autopsy (*n* = 46) and synchronous malignancies within a time interval of 3 months before or after MM diagnosis (*n* = 237) were excluded.

Using competing-risk-regression analysis, we estimated subdistribution hazard ratios (SHRs) with 95% confidence intervals (CIs). This technique accounts for death as a competing risk before SPM development. Two models were constructed. The first model (M1) included the binary variable of a PMD before MM diagnosis. In the second model (M2), patients with a PMD were classified as patients (a) with or (b) without receipt of systemic therapy and/or radiotherapy before MM diagnosis. In the absence of an SPM after the MM diagnosis or death, patients were censored at the time of emigration or at the end of the study (i.e., December 31, 2018). Both models were adjusted for age at MM diagnosis, sex, and calendar period of MM diagnosis. Overall survival (OS) was defined as the time between MM diagnosis and death from any cause. Mortality risk was calculated using Cox proportional-hazard models for M1 and M2. The complete methods are provided in the Supplemental. The Privacy Review Board of the NCR approved the use of anonymous data for this study.

In total, 18,030 MM patients were included, of whom 1489 (8.3%) with a PMD and 1334 (7.4%) with an SPM. Baseline characteristics at MM diagnosis are presented in Supplemental Table 1 according to the presence or absence of a PMD. Site-specific PMDs and SPMs are presented in Fig. 1 (panel A) and described in detail in the Supplemental.

**Fig. 1:**
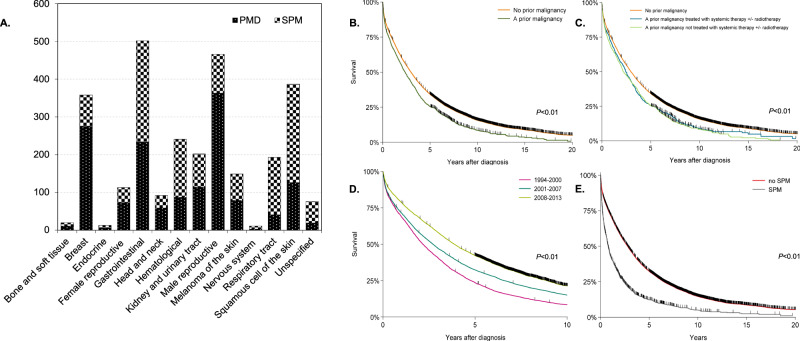
Prior (PMD) and subsequent primary malignancies (SPM), and overall survival (OS) among patients with multiple myeloma (MM). Panel **A** presents the site-specific numbers of prior and subsequent primary malignancies among patients with MM. OS curves are presented in panels **B**–**E**. Panel **B** presents Kaplan–Meier (KM) estimates of OS in which the exposure was the binary variable of a PMD before MM diagnosis (no vs. yes). Panel **C** presents KM estimates of OS in which patients with a PMD were classified as patients (i) with or (ii) without receipt of systemic or radiotherapy before MM diagnosis. Panel **D** presents KM estimates of OS in which patients were classified according to the calendar period of MM diagnosis, i.e., (i) 1994–2000, (ii) 2001–2007, and (iii) 2008–2013. Panel **E** presents KM estimates of OS in which patients were classified according to the presence or absence of an SPM following MM diagnosis. For patients without an SPM, OS is presented in years from MM diagnosis, and for patients with an SPM, OS is presented in years from the onset of an SPM. The *P*-value of the log-rank test is indicated in the KM figures.

The cumulative incidence of SPMs according to a PMD and calendar period of MM diagnosis is presented in Supplemental Fig. 1. Multivariable analysis revealed that a PMD was not associated with a higher SPM incidence (M1, Table 1A), irrespective of whether a PMD was treated with systemic therapy and/or radiotherapy or not (M2, Table 1A). In contrast, male sex and the most recent calendar period were independently associated with a greater cumulative incidence of SPMs. However, age shows opposing cumulative incidences of SPMs. More specifically, compared with patients aged 18–65 years, patients aged 66–70 years had a greater incidence of SPMs, whereas patients aged >70 years had a lower cumulative incidence of SPMs. The lower cumulative incidence of SPMs for patients aged >70 years may suggest that these patients more often die from other causes before the onset of SPMs. Site-specific analysis according to a PMD showed that having had a PMD was associated with a lower incidence of cancer of the male reproductive system and gastrointestinal tract (Supplemental Table 2). Moreover, regarding calendar period, we observed increased incidences of squamous-cell carcinomas of the skin and hematological malignancies over time, particularly myelodysplastic syndrome (MDS) and acute myeloid leukemia (AML, Table 1B).

**Table 1:** Competing risk regression models for the association between a history of malignancies and the development of subsequent primary malignancies after multiple myeloma patients in the Netherlands (panel **A**) and for risk of developing a certain site-specific subsequent malignancy among MM patients according to calendar period, using competing risk regression (panel **B**).

	Univariable			Multivariable					
				M1			M2		
	SHR	95% CI	*P*-value*	SHR	95% CI	*P*-value*	SHR	95% CI	*P*-value*
*Prior malignancy diagnosis*									
No	1	reference		1	reference				
Yes	0.98	0.80–1.20	0.85	0.97	0.79–1.18	0.73			
*Prior malignancy diagnosis*									
No	1	reference					1	reference	
Yes with ST and/or RT	0.95	0.71–1.29	0.76				0.94	0.69–1.28	0.70
Yes without ST and/or RT	1.00	0.78–1.29	0.99				0.98	0.76–1.27	0.89
*Period of MM diagnosis*									
1994–2000	1	reference		1	reference		1	reference	
2001–2007	1.26	1.10–1.45	**0.01**	1.25	1.09–1.44	**<0.01**	1.25	1.09–1.44	**<0.01**
2008–2013	1.39	1.21–1.59	**<0.01**	1.38	1.20–1.58	**<0.01**	1.38	1.20–1.58	**<0.01**
*Sex*									
Female	1	reference		1	reference		1	reference	
Male	1.40	1.25–1.56	**<0.01**	1.36	1.22–1.53	**<0.01**	1.36	1.22–1.53	**<0.01**
*Age at MM diagnosis (years)*									
18–65	1	reference		1	reference		1	reference	
66–70	1.18	1.00–1.39	**0.05**	1.19	1.01–1.40	**0.04**	1.19	1.01–1.40	**0.04**
>70	0.82	0.73–0.92	**<0.01**	0.84	0.75–0.95	**<0.01**	0.84	0.75–0.95	**<0.01**

Site-specific SPM^c^	Calendar period of MM diagnosis^a^								
	1994–2000			2001–2007			2008–2013		
	SHR	95% CI	*P*-value^b^	SHR	95% CI	*P*-value^b^	SHR	95% CI	*P*-value^b^
Breast (*n* = 82)	1	reference		**1.76**	**1.01–3.05**	**0.04**	1.31	0.73–2.35	0.37
Gastrointestinal (*n* = 269)	1	reference		1.33	0.98–1.80	0.06	1.14	0.84–1.55	0.41
Hematological (*n* = 154)	1	reference		**1.58**	**1.03–2.43**	**0.04**	**1.70**	**1.11–2.59**	**0.01**
MDS/AML (*n* = 81)	1	reference		**1.89**	**1.00–3.56**	**0.05**	**2.22**	**1.20–4.14**	**0.01**
Kidney and urinary tract (*n* = 88)	1	reference		0.97	0.56–1.68	0.92	1.23	0.73–2.06	0.43
Male reproductive (*n* = 103)	1	reference		1.20	0.74–1.96	0.46	1.08	0.66–1.77	0.76
Melanoma of the skin (*n* = 70)	1	reference		0.61	0.30–1.25	0.18	1.73	0.99–3.03	0.06
Respiratory tract (*n* = 152)	1	reference		1.01	0.67–1.53	0.95	1.23	0.83–1.82	0.31
Squamous cell of the skin (*n* = 261)	1	reference		**1.85**	**1.29–2.66**	**<0.01**	**2.44**	**1.73–3.45**	**<0.01**

*P-values are compared with the reference category. Statistically significant *P*-values (*P* < 0.05) are presented in bold.

*Abbreviations*: *M1* model 1, *M2* model 2, *CI* confidence interval, *MM* multiple myeloma, *SHR* subdistribution hazard ratio, *RT* radiotherapy, *ST* systemic therapy, *SHR* subdistribution hazard ratio, *CI* confidence interval, *SPM* subsequent primary malignancy, *MDS/AML* myelodysplastic syndrome/acute myeloid leukemia.

^a^This model is concurrently adjusted for sex, a primary malignancy diagnosis before MM diagnosis and age.

^b^*P*-values were compared to the reference category, statistically significant *p*-values (*P* < 0.05) are presented in bold.

^c^Analyses for site-specific subsequent primary malignancies that were rare (<5%) were omitted in this table.

In Figure 1, OS according to PMD (panels B and C), calendar period of MM diagnosis (panel D), and SPM (panel E) is presented. Multivariable assessment showed that patients with a PMD had an increased mortality risk compared with patients without a PMD (M1, Supplemental Table 3). This observation was irrespective of whether a PMD was treated with systemic therapy and/or radiotherapy or not (M2, Supplemental Table 3). Furthermore, age, male sex, and development of an SPM were independently associated with higher mortality risk, while mortality risk decreased in more recent calendar periods (Supplemental Table 3).

In this nationwide, population-based study, SPM incidence among MM patients increased over time, suggesting clues for MM therapy-related carcinogenesis rather than genetic susceptibility to cancer, since we did not find an association between a PMD and SPM development. Although survival improved markedly over time, MM patients with an SPM have an increased mortality risk.

With each increment of calendar period, used as a surrogate for the evolution of MM therapy, we observed an overall increase of SPMs, particularly squamous-cell carcinomas of the skin, MDS, and AML. This is in accordance with findings from clinical trials [1, 4], retrospective (population-based) studies [9–11], as well as with data discussed in a recently published review [8]. A potential mechanism may lie in the cumulative exposure to mutagenic anti-MM agents (e.g., alkylating drugs), thereby increasing the risk of subsequent MDS/AML development [11, 12]. Indeed, high-dose melphalan exposure in MM patients increases mutational burden following the end of first-line treatment, until relapse by approximately 10–20% [13]. In addition, a modest increase in hematological SPMs was reported in patients managed with lenalidomide-maintenance therapy when applied after ASCT or continuing after induction therapy [8]. Also, an SPM developed more rapidly in patients managed with lenalidomide. Another mechanism for the increased SPM risk involves immunosuppression, which may contribute to the development of squamous-cell carcinomas of the skin [14].

To further build upon potential etiologies of SPM development, we did not observe an association between PMDs and SPM incidence, as opposed to the results from a Swedish population-based study among 19,097 MM patients diagnosed during 1973 and 2010 [7]. Explanations for these opposing results may lie in the type of regression analysis applied or the in- and exclusion criteria applied for synchronous malignancies, benign, borderline, and in situ malignancies, and basal-cell carcinomas. Of note, we performed sensitivity analyses to confirm that our results were not dependent on the definition for synchronous malignancies (data not shown). The underlying mechanisms for our, somewhat surprisingly, inverse association with the male reproductive system and gastrointestinal system are unknown. This association might be driven by changes in the perception of MM from a rapidly fatal to a more chronic condition, thereby affecting screening practices for MM survivors. Future research with large numbers of site-specific SPMs compared with the general population is necessary to address this hypothesis.

By assessing the impact of an SPM on mortality risk, we observed a higher mortality risk among MM patients with a PMD than MM patients without a PMD. This finding might be explained by the (late) effects from prior systemic therapy and/or radiotherapy, potentially leading to organ dysfunction such as compromised bone marrow function and cardiovascular disease. Furthermore, the risk of mortality decreased over time, attributable to improved, more targeted, anti-MM therapy, and this decrease in mortality is in concordance with prior literature, including two meta-analyses [5, 15]. Collectively, MM patients benefited from the therapeutic advances achieved over the past. However, this finding is offset by an increased risk of mortality due to an SPM.

The main strength of our study is the use of comprehensive data available for individual patients from a long-running and well-established nationwide cancer registry, including the availability of use of treatment for PMDs. The limitations of our study pertain to the lack of information on therapy of a PMD beyond one year post diagnosis and detailed information on patient, MM, and treatment characteristics throughout most of the study period (i.e., 1989–2013). Consequently, we could not entirely rule out residual confounding. In addition, the number of patients with a PMD and an SPM was too limited to perform analyses within M2 for site-specific SPMs and other subgroup analyses. Last, patients without a PMD diagnosed before 1989 may have been misclassified due to left truncation since we only had a five-year lead time. Despite these limitations, cancer-registries remain the standard for cancer surveillance activities.

In summary, no association between a PMD and SPM development was observed among MM patients. However, although survival improved markedly over time, this finding is offset by an increased mortality risk due to an SPM. Therefore, augmented cancer surveillance is desired for early detection and appropriate SPM management, which, in turn, might reduce the impact of SPMs on the outcome. This recommendation is particularly relevant for patients managed with contemporary therapeutics for which, as yet, the effect on SPM development is ill-defined.

## Supplementary information

Supplemental
