# Supplementary material for: Increased mortality risk in multiple-myeloma patients with subsequent malignancies: a population-based study in the Netherlands
Source: Blood Cancer J. 2022 Mar 15;12(3):41. doi: 10.1038/s41408-022-00640-6 (PMC8924210; doi:10.1038/s41408-022-00640-6)
Supplement: Supplementary file 1 — Supplemental [file 41408_2022_640_MOESM1_ESM.docx]

**ONLINE APPENDIX**

**TITLE**

Increased mortality risk in multiple myeloma patients with subsequent malignancies: a population-based study in the Netherlands

**SUPPLEMENTAL METHODS**

**The Netherlands Cancer Registry**

Nationwide since 1989, the population-based Netherlands Cancer Registry (NCR), which is maintained and hosted by the Netherlands Comprehensive Cancer Organisation (IKNL), covers at least 95% of all malignancies in the Netherlands^1^. All newly diagnosed malignancies in the Netherlands are reported to the NCR via the Nationwide Network of Histopathology and Cytopathology, and the National Registry of Hospital Discharges (i.e. inpatient and outpatient discharges). Information on dates of birth and diagnosis, sex, disease topography and morphology, and first-line therapy is routinely recorded in the NCR by trained registrars of the NCR through retrospective medical records review. Topography and morphology are coded according to the International Classification of Diseases for Oncology (ICD-O), of which the morphology code 9732 was used in the current study for identifying patients with multiple myeloma (MM). Information on the last known vital status for all patients (i.e. alive, death, or emigration) is obtained through annual linkage with the Nationwide Population Registries Network that holds vital statistics on all residents in the Netherlands.

Prior malignancy diagnosis (PMD) diagnosed between January 1, 1989, and December 31, 2013, and subsequent primary malignancies (SPM) diagnosed between January 1, 1994, and December 31, 2018, were identified as site-specific malignancies using the third edition of the International Classification of Diseases for Oncology, *i.e.* bone and soft tissue; breast; endocrine; hematologic; (fe)male reproductive; gastrointestinal; head and neck; kidney and urinary tract; melanoma of the skin; nervous system; respiratory tract; squamous cell of the skin; unspecified. Therapy for PMDs was defined as receipt of systemic therapy and/or radiotherapy and no receipt of systemic therapy and/or radiotherapy.

**Statistical analyses**

Descriptive statistics were employed to compare patient characteristics between those with and without an SPM. The Pearson chi-square test was applied to compare categorical variables. The Mann-Whitney U test was used to compare continuous variables. The sub-distribution hazard ratio (SHR) describes the relative change in the instantaneous rate of the occurrence of an SPM in those who did not develop an SPM during follow-up (i.e., the event of interest) and those who died before that event occurred (i.e., the competing risk). Given the relationship with cumulative incidence function for the distribution hazard function, the SHRs can also be interpreted as the effect of a PMD on the incidence of SPMs. Of note, the magnitude of the effect of a PMD on the incidence of SPMs cannot be directly quantified by using SHRs. We constructed two competing risk regression models as per the Fine and Gray methodology^2^ to estimate SHRs with 95% confidence intervals (CIs). In Model 1 (M1), the exposure was the binary variable of a PMD before MM diagnosis (no *versus* yes). In Model 2 (M2), patients with a PMD were classified as patients (a) with or (b) without receipt of systemic therapy and/or radiotherapy before the diagnosis of MM. Consequently, effect estimates for PMD as a categorical variable might provide clues on the effect of sequelae of prior cancer therapy on SPM development. The categorization of three calendar periods of MM diagnosis (1994-2000, 2001-2007, and 2008-2013) was based on the implementation of SCT (≥2001) and first-generation novel agents such as IMiDs (≥2008) into treatment algorithms for patients with multiple myeloma (MM) in the Netherlands and therefore calendar periods might provide clues on the effect of continuous MM treatment changes on SPM development. SPM diagnosis was a dichotomous outcome variable, and death without SPM was the competing event. In the absence of an event, patients were censored at the time of emigration or at the end of the study (i.e., December 31, 2018), whichever occurred first. M1 and M2 were additionally adjusted for age at MM diagnosis, sex, and calendar period of MM diagnosis. SHRs for the association between a PMD and the diagnosis of first SPM were also calculated for site-specific SPMs by using M1.

OS was defined as the time between MM diagnosis and death from any cause. Survival distributions were compared with the log-rank test. Similar to the competing risk models, we constructed two Cox proportional hazard models to calculate hazard ratios (HRs) with 95% CIs for the association between a PMD and mortality, and between treatment practices of MM and mortality. Here, patients were censored at the time of emigration or at the end of the study (i.e. December 31, 2018), whichever occurred first. The risk of mortality was estimated using the similar confounders of the competing risk regression models in addition to the diagnosis of SPM as a time-varying covariate.

A *P*<0.05 indicated statistical significance. All statistical analyses were performed with STATA Statistical Software Release 17.0 (College Station, TX, USA).

**Supplemental references**

1. Schouten LJ, Hoppener P, van den Brandt PA, Knottnerus JA, Jager JJ. Completeness of cancer registration in Limburg, The Netherlands. *Int J Epidemiol*. 1993;22(3):369-376.

2. Fine JP, Gray RJ. A proportional hazard model for the subdistribution of a competing risk. *J Am Statist Assoc*. 1999;94(446):496-509.

**Supplemental results**

**Supplemental Table 1.** Patient characteristics

|  | **MM with a PMD** | **MM without a PMD** | **Total** |
| --- | --- | --- | --- |
| **Patients, n (% row)** | 1,489 (8.3) | 16,541 (91.7) | 18,030 |
| **Patients with SPM, n (%)** | 107 (7.2) | 1,227 (7.4) | 1,334 (7.4) |
| **Age at MM diagnosis, median (IQR), years** | 75.7 (69.0-80.7) | 70.2 (61.2-77.7) | 70.7 (61.7-78.0) |
| **Male sex, n (%)** | 883 (59.3) | 8.939 (54.0) | 9,822 (54.5) |
| **Calendar period of MM diagnosis, n (%)** |  |  |  |
| 1994-2000, pre-ASCT period | 268 (18.0) | 5,003 (30.3) | 5,271 (29.2) |
| 2001-2007, pre-novel agents period | 480 (32.2) | 5,561 (33.6) | 6,041 (33.5) |
| 2008-2013, novel agents period | 741 (49.8) | 5,977 (36.1) | 6,718 (37.3) |
| **Year of MM diagnosis, median (IQR)** | 2007 (2003-2011) | 2005 (1999-2009) | 2005 (2000-2010) |
| **Median time from first PMD to MM, years (IQR)** |  |  |  |
| Total | 5.5 (2.7-9.7) | - | - |
| With an SPM | 5.8 (2.8-10.7) | - | - |
| Without an SPM | 5.5 (2.7-9.7) | - | - |
| **Median follow-up time after MM diagnosis, years (IQR)** | 2.4 (0.6-5.1) | 3.0 (0.9-6.2) | 2.9 (0.9-6.1) |

Abbreviations: MM, multiple myeloma; IQR, interquartile range; PMD, prior malignancy diagnosis; SPM, subsequent primary malignancy; ASCT, autologous stem cell transplantation

**Supplemental Table 2.** The subdistribution hazard ratio and corresponding confidence intervals for the risk of developing a certain site-specific subsequent malignancy among MM patients, according to prior malignancy diagnosis (compared with those without) using competing risk regression.

| **Site-specific SPM^c^** | | **Primary Malignancy Diagnosis^a^** | | | | | | |
| --- | --- | --- | --- | --- | --- | --- | --- | --- |
|  |  | **No** | | | **Yes** | | | |
|  |  | **SHR** | **95% CI** | ***P*-value^b^** | **SHR** | **95% CI** | ***P*-value^b^** | |
| **Breast (n = 82)** |  | 1 | *reference* |  | 0.35 | 0.09-1.45 | 0.15 | |
| **Gastrointestinal (n = 269)** |  | 1 | *reference* |  | **0.57** | **0.33-0.99** | **0.04** | |
| **Haematological (n = 154)** |  | 1 | *reference* |  | 0.85 | 0.44-1.64 | 0.63 | |
| **MDS/AML (n = 81)** |  | 1 | *reference* |  | 1.07 | 0.47-2.43 | 0.88 | |
| **Kidney and urinary tract (n = 88)** |  | 1 | *reference* |  | 1.12 | 0.55-2.27 | 0.75 | |
| **Male reproductive (n = 103)** |  | 1 | *reference* |  | **0.09** | **0.01-0.66** | **0.02** | |
| **Melanoma of the skin (n = 70)** |  | 1 | *reference* |  | 1.97 | 0.97-4.00 | 0.06 | |
| **Respiratory tract (n = 152)** |  | 1 | *reference* |  | 1.17 | 0.67-2.05 | 0.57 | |
| **Squamous cell of the skin (n = 261)** |  | 1 | *reference* |  | 1.13 | 0.75-1.71 | 0.56 | |
| Abbreviations: SHR, sub-distribution hazard ratio; CI, confidence interval; SPM, subsequent primary malignancy; MDS/AML, myelodysplastic syndrome/acute myeloid leukemia. | | | | | | | | |
| ^a^This model is concurrently adjusted for sex, calendar period of diagnosis and age. | | | | | | | | |
| ^b^*P*-values were compared to the reference category, statistically significant *p*-values (*P*<0.05) are presented in bold.  ^c^Analyses for site-specific subsequent primary malignancies that were rare (<5%) were omitted in this table. | | | | | | | |  |

**Supplemental Table 3.** Cox regression models for the association between a history of malignancies and

mortality among multiple myeloma patients in the Netherlands.

|  | **Univariable** | | | **Multivariable** | | | | | |
| --- | --- | --- | --- | --- | --- | --- | --- | --- | --- |
|  |  |  |  | **M1** |  |  | **M2** | | |
|  | HR | 95% CI | *P*-value*** | HR | 95% CI | *P*-value*** | HR | 95% CI | *P*-value* |
| **Prior malignancy diagnosis** |  |  |  |  |  |  |  |  |  |
| No | *1* | *reference* |  | *1* | *reference* |  |  |  |  |
| Yes | 1.26 | 1.19-1.33 | ***<0.01*** | 1.13 | 1.07-1.19 | ***<0.01*** |  |  |  |
| **Prior malignancy diagnosis** |  |  |  |  |  |  |  |  |  |
| No | *1* | *reference* |  |  |  |  | *1* | *reference* |  |
| Yes with ST and/or RT | 1.22 | 1.12-1.33 | ***<0.01*** |  |  |  | 1.11 | 1.02-1.21 | ***0.01*** |
| Yes without ST and/or RT | 1.29 | 1.20-1.38 | ***<0.01*** |  |  |  | 1.14 | 1.06-1.23 | ***<0.01*** |
| **Period of MM diagnosis** |  |  |  |  |  |  |  |  |  |
| 1994 - 2000 | *1* | *reference* |  | *1* | *reference* |  | *1* | *reference* |  |
| 2001 - 2007 | 0.78 | 0.75-0.81 | ***<0.01*** | 0.79 | 0.76-0.82 | ***<0.01*** | 0.79 | 0.76-0.82 | ***<0.01*** |
| 2008 - 2013 | 0.59 | 0.57-0.62 | ***<0.01*** | 0.58 | 0.56-0.61 | ***<0.01*** | 0.58 | 0.56-0.61 | ***<0.01*** |
| **Sex** |  |  |  |  |  |  |  |  |  |
| Female | *1* | *reference* |  | *1* | *reference* |  | *1* | *reference* |  |
| Male | 1.01 | 0.98-1.05 | *0.36* | 1.10 | 1.06-1.13 | ***<0.01*** | 1.10 | 1.06-1.13 | ***<0.01*** |
| **Age at MM diagnosis (years)** |  |  |  |  |  |  |  |  |  |
| 18-65 | 1 | reference |  | 1 | reference |  | 1 | reference |  |
| 66-70 | 1.63 | 1.55-1.73 | ***<0.01*** | 1.63 | 1.55-1.73 | ***<0.01*** | 1.63 | 1.55-1.73 | ***<0.01*** |
| >70 | 2.38 | 2.30-2.47 | ***<0.01*** | 2.40 | 2.31-2.49 | ***<0.01*** | 2.40 | 2.31-2.49 | ***<0.01*** |
| **Subsequent primary malignancy** |  |  |  |  |  |  |  |  |  |
| No | *1* | *reference* |  | *1* | *reference* |  | *1* | *reference* |  |
| Yes | 1.13 | 1.06-1.20 | ***<0.01*** | 1.22 | 1.14-1.30 | ***<0.01*** | 1.22 | 1.14-1.30 | ***<0.01*** |

*P-values are compared with the reference category. Statistically significant P-values (P<0.05) are presented in bold.

Abbreviations: M1, model 1; M2, model 2; CI, confidence interval; MM, multiple myeloma; HR, hazard ratio

**Supplemental** **Figure legend**

**Supplemental Figure 1.** Cumulative incidence function of subsequent primary malignancies after multiple myeloma. In panel **A**, the exposure was the binary variable of a PMD before MM diagnosis (yes versus no). In panel **B**, patients with a PMD were classified as patients (i) with or (ii) without receipt of systemic therapy or radiotherapy before MM diagnosis. In panel **C**, cumulative incidence function is presented according to calendar period of MM diagnosis (i) 1994-2000, (ii) 2001-2007, and (iii) 2008-2013.

**A.**

**B.**

**C.**
